# Local topology and bifurcation hot-spots in proteins with SARS-CoV-2 spike protein as an example

**DOI:** 10.1371/journal.pone.0257886

**Published:** 2021-09-30

**Authors:** Xubiao Peng, Antti J. Niemi

**Affiliations:** 1 Center for Quantum Technology Research and Key Laboratory of Advanced Optoelectronic Quantum Architecture and Measurements (MOE), Beijing Institute of Technology, Beijing, China; 2 Nordita, Stockholm University, Stockholm, Sweden; 3 School of Physics, Beijing Institute of Technology, Beijing, China; 4 Pacific Quantum Center and School of Biomedicine, Far Eastern Federal University, Vladivostok, Russia; Russian Academy of Medical Sciences, RUSSIAN FEDERATION

## Abstract

Novel topological methods are introduced to protein research. The aim is to identify hot-spot sites where a bifurcation can alter the local topology of the protein backbone. Since the shape of a protein is intimately related to its biological function, a substitution that causes a bifurcation should have an enhanced capacity to change the protein’s function. The methodology applies to any protein but it is developed with the SARS-CoV-2 spike protein as a timely example. First, topological criteria are introduced to identify and classify potential bifurcation hot-spot sites along the protein backbone. Then, the expected outcome of asubstitution, if it occurs, is estimated for a general class of hot-spots, using a comparative analysis of the surrounding backbone segments. The analysis combines the statistics of structurally commensurate amino acid fragments in the Protein Data Bank with general stereochemical considerations. It is observed that the notorious D614G substitution of the spike protein is a good example of a bifurcation hot-spot. A number of topologically similar examples are then analyzed in detail, some of them are even better candidates for a bifurcation hot-spot than D614G. The local topology of the more recently observed N501Y substitution is also inspected, and it is found that this site is proximal to a different kind of local topology changing bifurcation.

## Introduction

Topology, as the terminology is used in mathematical literature, addresses properties of a geometric object that are preserved under continuous deformations. Topology based methods are among the most versatile tools available to predict, model and analyze a wide range of Physics related phenomena, from fundamental interactions to condensed matter and dynamical systems [1–4]. However, despite the apparently rich and intriguing topology of many biomolecules, thus far topological methods have been sparsely applied to biophysical problems. Among the notable exemptions are the analysis of enzyme action on DNA and the challenge to understand the biological role of knots in folded proteins [5]; here we do not consider topology as it is defined in structural biology, where the concept relates to orientation of regular secondary structures in a protein backbone.

Here a novel topological methodology, apparently with no previous physical or biological applications, is introduced and developed for biophysical purposes. The methodology builds on the concept of a bifurcation [4, 6] that can change the local topology of a protein backbone. The mathematical framework was originally introduced and analyzed by Arnol’d [7–9]. He investigated continuously differentiable space curves, and the present article adapts his results to the case of a discrete piecewise linear chain such as the protein C*α* backbone. Bifurcation theory describes how a small change in an input parameter can cause a large scale change in the system [4, 6], bifurcations are a common cause for qualitative changes in a physical system. Accordingly it is proposed here that in the case of a protein backbone, amino acid sites that are proximal to a local topology changing bifurcation are good candidates for a hot-spot where a small alteration *e.g*. by substitution can have a large biological effect.

The methodology is very general, it should have value to a wide range of future investigations. In particular, it is applicable to any protein structure, even though it is presented here with the spike protein of the severe acute respiratory syndrome coronavirus 2 (SARS-CoV-2) as an example. The choice reflects the current urgency to understand the function of the virus that causes COVID-19, a global public health emergency that continues to spread across the world.

**Fig 1:**
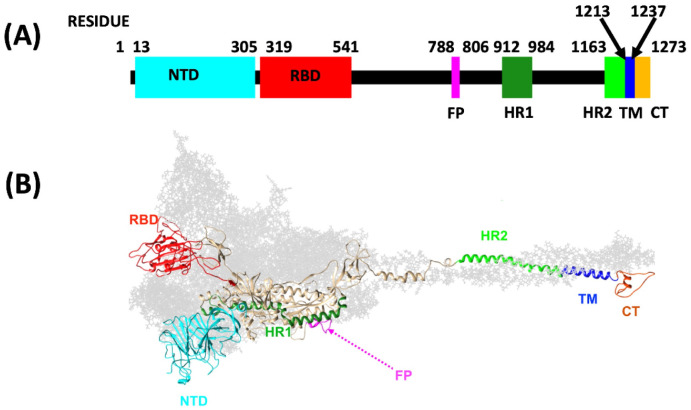
(A) Spike protein subunit S1 consists of the N-terminal domain NTD and the receptor binding domain RBD. The subunit S2 consists of the fusion peptide FP, two heptapeptide repeat sequences HR1 and HR2, the transmembrane domain TM and the cytoplasm domain tail CT. (B) A three-dimensional model of the full length closed conformation spike protein, based on the PDB structure 6VXX (closed state) and adapted from [30].

Several studies of the SARS-CoV-2 virus have been published including investigations on the source of infection [10–14], the mechanism of transmission [15–19], and the structure and function of its various proteins [20–26]. These studies also detail the biophysical and biochemical properties of the spike protein, a transmembrane glycoprotein that assembles into a homo-trimer to cover the virion surface and gives the virus its distinctive crown-like look. For the present purposes the following short introduction to the spike protein structure and function is sufficient:

The spike protein has three different conformations. The prefusion conformation, the intermediate conformation, and the post-fusion conformation. Before the fusion with a host cell takes place the spike protein is in the prefusion conformation, and the present study focuses solely on the protein in this conformation. The monomer structure of the prefusion conformation is as follows: Starting from the N-terminal, first there is a short signal peptide. This is followed by two larger subunit, S1 and S2. The subunit S1 can recognize the host cell and bind to the receptor angiotensin-converting enzyme 2 (ACE2). The subunit S2 can bind to the membrane of the host cell, to mediate the fusion between the virus and the host cell. The Fig 1A identifies the major functional domains in these subunits in a monomeric spike protein [20, 21]: The S1 subunit consists of residues between the sites 14-685. It starts with a N-terminal domain (NTD) with residues 14-305 [27]. The NTD is followed by the receptor binding domain (RBD, residues 319-541) [28]. The junction segment between the S1 and S2 subunits includes several cleavage sites [29]. The S2 subunit that comprises the rest of the protein, contains the fusion peptide (FP, residues 788-806) followed by two heptapeptide repeat sequences (HR1, residues 912-984) and (HR2, residues 1163-1213), the transmembrane domain (TM, residues 1213-1237) and the cytoplasm domain tail (CT, residues 1237-1273) [30].

In the prefusion conformation the protein has two principal conformational states, called the closed state and the open state, and the Fig 1B shows the protein in the closed state. When the virus starts interacting with the host cell the spike protein transits from the closed state to the open state, with a major conformational change that takes place in the S1 subunit [22].

The spike protein has a pivotal role in processes that range from receptor recognition to viral attachment and entry into host cell. It is a major target in both vaccine research and therapeutic research that combat the SARS-CoV-2 virus. But the spike protein evolves and mutates continuously which makes it a demanding target for the development of antiviral inhibitors. Among the prominent examples of a mutation in the spike protein, which is analyzed here as an example of a bifurcation, is the D→G substitution that occurred at its site 614, near the junction between the subunits S1 and S2. Apparently this mutation converted SARS-CoV-2 into a more transmissible form, and the D614G mutated virus now dominates in the global COVID-19 pandemic [31]. Another, more recent mutation that is also analyzed here as an example of a bifurcation is the N→Y substitution that occurred at site 501 in the RDB domain [32].

The Methods section first summarizes the known mathematical results on geometry and local topology of a smooth space curve. The focus is on the two curve specific bifurcations that can alter the local topology of a curve. These bifurcations were introduced by Arnol’d [7–9] who called them the inflection point perestroika and the bi-flattening perestroika. The Methods section adapts these bifurcations to the case of a piecewise linear chain such as the generic protein C*α* backbone.

The Results section explains in detail how to apply the general methodology to identify and classify bifurcation hot-spots, in the case of an actual protein backbone; the SARS-CoV-2 spike protein is used as a timely example but any protein backbone can be analyzed in the same manner. For the spike protein, the Protein Data Bank structures 6VXX (closed state) 6VYB (open state) [22] and 6XS6 (D614G mutation) [33] are used. First, a general class of local topologies with a bifurcation hot-spot is identified. It consists of backbone segments that surround a site that is proximal to a flattening point: The D614G substitution occurred at such a hot-spot, thus the details of the methodology are worked out with the site 614 of the spike protein as an example. All similar bifurcation hot-spot sites of the spike protein, with a site proximal to a flattening point, are tabulated in the currently available PDB structures. Additional examples are analyzed, including the identification of the likely amino acid substitution that may take place at such a hot-spot. Finally, the site of the N501Y substitution is identified as a different kind of hot-spot, and analyzed as an additional example of a bifurcation.

## Methods

### Local topology of regular space curves

The bifurcations that can change the local topology of a space curve were introduced and analyzed by Arnol’d in a series of articles [7–9]; see also [34, 35]. For an everyday example, one can think of an entangled phone cord: Commonly, the tangles are not removable by small local deformations of the cord. For that, the cord needs to be bent and twisted in a manner that changes its local topology, by a series of bifurcations. This subsection summarizes the main results in [7–9, 34, 35] and sets the stage for understanding the local topology and bifurcations in the case of protein C*α* backbone, which is the subject of the next subsection.

The starting point is in the Frenet framing that governs the geometry of a regular, analytic space curve that is not a straight line [36]. To describe this framing, consider a parametric representation $x\left(s\right)\in R^{3}$ of a space curve, with *s* ∈ [0, *L*] the arc-length parameter and *L* its fixed length. The unit length tangent vector is

$$\begin{array}{c} t=\frac{dx(s)}{ds}\equiv x_{s} \end{array}$$ (1)

The unit length binormal vector is

$$\begin{array}{c} b=\frac{x_{s}\times x_{ss}}{|x_{s}\times x_{ss}|}\;\;\;\;\;\;\;\left(x_{ss}\equiv \frac{d^{2}x\left(s\right)}{ds^{2}}\right) \end{array}$$ (2)

and the unit length normal vector is

$$\begin{array}{c} n=b\times t \end{array}$$ (3)

Together, the three vectors (**n**(*s*), **b**(*s*), **t**(*s*)) define the right-handed orthonormal Frenet frame at the regular point **x**(*s*) of the curve. They govern the geometry of the curve in terms of curvature *κ*(*s*)

$$\begin{array}{c} \kappa \left(s\right)=n\cdot \;\frac{d}{ds}t \end{array}$$ (4)

that describes how the curve bends on the osculating plane spanned by **t**(*s*) and **n**(*s*), and torsion *τ*(*s*)

$$\begin{array}{c} \tau \left(s\right)=-n\cdot \;\frac{d}{ds}b \end{array}$$ (5)

that measures how the curve deviates from this osculating plane. The Eqs (1)–(5) are summarized by the Frenet equation [36]:

$$\begin{array}{c} \frac{d}{ds}(\begin{array}{c} n\\ b\\ t \end{array})\;=\;(\begin{array}{ccc} 0 & \tau & -\kappa \\ -\tau & 0 & 0\\ \kappa & 0 & 0 \end{array})(\begin{array}{c} n\\ b\\ t \end{array}) \end{array}$$ (6)

The Frenet frames can be introduced whenever the curvature *κ*(*s*) is non-vanishing. In the specific limit where the curvature is very small in comparison to the torsion, but does not vanish

$$|\frac{\kappa (s)}{\tau (s)}|\to 0$$

the frames obey

$$\begin{array}{cc} \frac{d}{ds}\left(n+ib\right) & \approx \;-\;i\tau (n+ib)\\ \frac{d}{ds}t & ∼\;0 \end{array}$$ (7)

This limit, that not discussed in [7–9, 34, 35] but turns out to be relevant to protein backbones, describes a framed, straight line with framing that rotates around the line at a rate and direction that is determined by *τ*(*s*).

In the following it is assumed that the curve is open and its shape can change freely by local deformations, but the end points are always fixed. The shape changes are governed by the following rules [7–9, 34, 35]:

At a point where the curvature *κ*(*s*) vanishes, the Frenet frame can not be defined; here it is assumed that *κ*(*s*) has only isolated simple zeroes. The points where *κ*(*s*) = 0 are called the inflection points of the curve. A local deformation that retains the osculating plane can move an inflection point along the curve. But if a deformation lifts a point with *κ*(*s*) = 0 off the osculating plane the inflection point becomes removed. This implies that the co-dimension of an inflection point is two: An inflection point is not a local topological invariant of the curve, and a generic curve does not have any inflection points.

A point where the torsion vanishes *τ*(*s*) = 0 is a flattening point. Unlike an inflection point, at least one single simple flattening point is generically present in a curve. A single simple flattening point is a local topological invariant that can not be removed by any continuous local deformation of the curve, it can only be moved along the curve.

The three vectors (**n**(*s*), **b**(*s*), **t**(*s*)) determine a framing of the curve, and either **b**(*s*) or **n**(*s*) or their linear combination can be chosen as the framing vector. The self-linking of the curve describes how it links with a nearby curve that is obtained by pushing points of the original curve along the framing vector. The self-linking number is a local topological invariant of the curve, in the absence of an inflection point the self-linking number can not change.

When the shape of a curve changes freely, an isolated inflection point generically occurs at some instance. When an inflection point appears the curve undergoes a bifurcation that is called an inflection point perestroika [7–9]. This bifurcation can change he number of flattening points: Since the torsion *τ*(*s*) changes its sign at a simple flattening point, and since the curvature *κ*(*s*) is generically not zero, an inflection point perestroika commonly changes the number of simple flattening points by two.

When the shape of a curve changes so that a pair of flattening points comes together they combine into a single bi-flattening point. A bi-flattening point can then be removed by a further, generic local deformation of the curve. Similarly, a bi-flattening point can first be created by a proper local deformation of a curve, and when the curve is further deformed the bi-flattening point can resolve into two separate simple flattening points. When either of these occur the curve undergoes a bifurcation that is called a bi-flattening perestroika [7–9].

Inflection point perestroika and bi-flattening perestroika are the only two bifurcations where the number of flattening points can change. Furthermore, according to [7–9, 34, 35]the number of flattening points and the self-linking number that is determined by the Frenet framing are the only two curve specific local topological invariants that can be assigned to a curve.

The number of flattening points and the self-linking number are independent topological invariants, but in the presence of an inflection point they can interfere with each other. For example, when a curve is deformed so that two simple flattening points become combined and disappear in a bi-flattening perestroika, the self-linking number in general does not change. However, if the bi-flattening perestroika occurs in conjunction of an inflection point perestroika the self-linking number can change: If the torsion is initially positive and two flattening points combine and disappear with the passage of an inflection point, the self-linking number increases by one. But if the torsion is initially negative the self-linking number decreases by one.

Note that the limiting case (7) is excluded in these analyses but it will become important in the sequel, in applications to protein backbones.

### The geometry and local topology of a protein C*α* backbone

The relations that are described in the previous subsection are valid for (thrice) continuously differentiable curves. In this subsection they are adapted to the case of a C*α* backbone that determines a piecewise linear polygonal chain, with C*α* atoms at the vertices.

Various shape changes are common in a biologically active protein. A polygonal chain such as the C*α* backbone can always be thought as a limiting case of a regular, analytic space curve. Thus the changes in the shape of the C*α* backbone are subject to same rules that govern the local topology of any regular space curve. In particular, the three essential shape deformations that can change the local topology *i.e*. discrete variants of inflection point perestroikas, bi-flattening perestroikas, and changes in a self-linking number should all have a profound role in physiological processes.

The discrete Frenet frame formalism is developed in [37]. The formalism describes the geometry of a piecewise linear chain with vertices **r**_*i*_ (*i* = 1, …, *N*) that in the case of a protein backbone are the space coordinates of the C*α* atoms. A line segment defines the unit tangent vector

$$\begin{array}{c} t_{i}=\frac{r_{i+1}-r_{i}}{|r_{i+1}-r_{i}|} \end{array}$$ (8)

It points from the center of the *i*^*th*^ C*α* atom towards the center of the (*i* + 1)^*st*^ C*α* atom. The unit binormal vector is

$$\begin{array}{c} b_{i}=\frac{t_{i-1}\times t_{i}}{|t_{i-1}\times t_{i}|} \end{array}$$ (9)

and the unit normal vector is

$$\begin{array}{c} n_{i}=b_{i}\times t_{i} \end{array}$$ (10)

The orthonormal triplet (**n**_*i*_, **b**_*i*_, **t**_*i*_) defines a discrete version of the Frenet frames (1)–(3) at each position **r**_*i*_ along the chain. In lieu of the curvature *κ*(*s*) and the torsion *τ*(*s*) there are now their discrete versions, the bond angles *κ*_*i*_ and the torsion angles *τ*_*i*_. The values of these angles can be computed from the discrete Frenet frames. The bond angles are

$$\begin{array}{c} \kappa_{i+1,i}\;=\;\text{arccos}\;(t_{i+1}\cdot t_{i})\;\equiv \;\kappa_{i+1}\left(r_{i},r_{i+1},r_{i+2}\right) \end{array}$$ (11)

and the torsion angles are

$$\begin{array}{c} \tau_{i+1,i}\;=\;\text{sign}\left\{b_{i}\times b_{i+1}\cdot t_{i}\right\}\cdot \text{arccos}\;(b_{i+1}\cdot b_{i})\;\equiv \;\tau_{i+1}\left(r_{i-1},r_{i},r_{i+1},r_{i+2}\right) \end{array}$$ (12)

It is notable that the value of the bond angle *κ*_*i*_ is evaluated from three, and the value of the torsion angle *τ*_*i*_ is evaluated from four consecutive vertices.

Conversely, when the values of the bond and torsion angles are all known, the discrete Frenet equation [37]

$$\begin{array}{c} \left(\begin{array}{c} n_{i+1}\\ b_{i+1}\\ t_{i+1} \end{array})={\left(\begin{array}{ccc} \text{cos}\;\kappa \;\text{cos}\;\tau & \text{cos}\;\kappa \;\text{sin}\;\tau & -\text{sin}\;\kappa \\ -\text{sin}\;\tau & \text{cos}\;\tau & 0\\ \text{sin}\;\kappa \;\text{cos}\;\tau & \text{sin}\;\kappa \;\text{sin}\;\tau & \text{cos}\;\kappa \end{array}\right)}_{\;i+1,i}(\begin{array}{c} n_{i}\\ b_{i}\\ t_{i} \end{array}\right) \end{array}$$ (13)

computes the Frenet frame at the vertex **r**_*i*+2_ from the frame at the preceding vertex **r**_*i*+1_; the entire chain can then be reconstructed in terms of these angles, up to a global rotation and translation [37]. Comparison of (6) and (13) shows that in a continuum limit [37] the discrete Frenet equation becomes the continuum Frenet equation [36].

**Fig 2:**
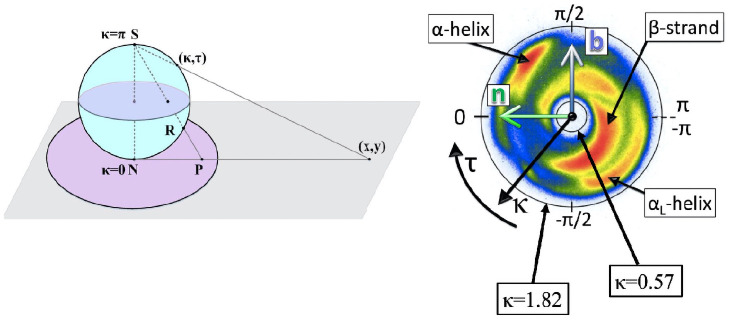
a) Stereographic projection (14) of two sphere from the north pole (N) with latitude *κ* and longitude *τ*, b) The stereographically projected Frenet frame map of backbone C*α* atoms, with major secondary structures identified. Also shown is the directions of the Frenet frame normal vector **n** and bi-normal vector **b**; the vector **t** points upwards from the figure. Colour coding corresponds to the number of PDB entries with red large, blue small and white none.

The fundamental range of the bond angles is *κ*_*i*_ ∈ [0, *π*] and in the case of the torsion angles *τ*_*i*_ ∈ [−*π*, *π*). For visualization purposes, the bond angles *κ*_*i*_ can be identified with the latitude angle of a two-sphere that is centered at the *i*^*th*^ C*α* atom; the north pole coincides with the inflection point *κ*_*i*_ = 0. The torsion angles *τ*_*i*_ ∈ [−*π*, *π*) correspond to the longitudinal angle on the sphere, the value increases in the counterclockwise direction around the tangent vector and the value *τ*_*i*_ = 0 of flattening points coincides with the great semi circle that starts from north pole and passes through the tip of the normal vector **n** to the south pole. The sphere can be stereographically projected onto the complex (*x*, *y*) plane. A projection from the south pole is

$$\begin{array}{c} z=x+iy\;\equiv \;\sqrt{x^{2}+y^{2}}\;e^{i\tau }\;=\;\text{tan}(\kappa /2)\;e^{i\tau } \end{array}$$ (14)

as shown in Fig 2: The north pole *i.e*. the point of inflection with *κ* = 0 becomes mapped to the origin (*x*, *y*) = (0, 0) and the south pole *κ* = *π* is sent to infinity.

The C*α* backbone can be visualized on the stereographically projected two sphere as follows [38]: At each C*α* atom, introduce the corresponding discrete Frenet frames (8)–(10). The base of the *i*^*th*^ tangent vector **t**_*i*_ that is located at the position **r**_*i*_ of the *i*^*th*^ C*α* atom coincides with the centre of a two-sphere with the vector **t**_*i*_ pointing towards its north pole. Now, translate the sphere from the location of the *i*^*th*^ C*α* atom to the location of the (*i* + 1)^*th*^ C*α* atom, without any rotation of the sphere with respect to the *i*^*th*^ Frenet frames. Identify the direction of **t**_*i*+1_, *i.e*. the direction towards the C*α* atom at site **r**_*i*+2_ from the site **r**_*i*+1_, on the surface of the sphere in terms of the ensuing spherical coordinates (*κ*_*i*_, *τ*_*i*_). When this construction is repeated for all the protein structures in Protein Data Bank that have been measured with better than 1.5 Å resolution, the result can be summarized by the map that is shown in Fig 2b). The color intensity correlates directly with the statistical distribution of the (*κ*_*i*_, *τ*_*i*_); red is large, blue is small and white is none. The map describes the direction of the C*α* carbon at **r**_*i*+2_ as it is seen at the vertex **r**_*i*+1_, in terms of the Frenet frames at **r**_*i*_.

Approximatively, the statistical distribution in Fig 2b) is concentrated within an annulus that corresponds to the latitude angle values (in radians) *κ* = 0.57 and *κ* = 1.82 shown in the Figure. The exterior of the annulus is a sterically excluded region. The entire interior is sterically allowed, but there are very few entries in this region. The four major secondary structure regions, *α*-helices, *β*-strands, left-handed *α*-helices and loops, are identified according to their PDB classification. For example, (*κ*, *τ*) values (in radians) for which

$$\begin{array}{c} \left\{ \;\begin{array}{ccc} \kappa_{i} & \approx & \frac{\pi }{2}\\ \tau_{i} & \approx & 1 \end{array}\right. \end{array}$$ (15)

describes a right-handed *α*-helix, and values for which

$$\begin{array}{c} \left\{ \;\begin{array}{ccc} \kappa_{i} & \approx & 1\\ \tau_{i} & \approx & \pm \pi \end{array}\right. \end{array}$$ (16)

describes a *β*-strand.

In the case of a regular space curve both *κ*(*s*) and *τ*(*s*) are smooth functions and the inflection points and the flattening points are easily identified as the points where *κ*(*s*) = 0 and *τ*(*s*) = 0. In a crystallographic protein structure where the C*α* positions are experimentally determined, an inflection point is detectable as a very small value of the bond angle *κ*_*i*_ at the proximal C*α* vertex. Similarly, the presence of a simple flattening point can be deduced from a very small torsion angle value at the proximal vertex, accompanied by a sign change in *τ* between two neighboring vertices; if the sign of *τ* does not change the proximal vertex has the character of a bi-flattening point. Accordingly, when searching for C*α* atoms where essential shape changes such as inflection point or bi-flattening perestroikas can take place, the natural points to start are the neighborhoods of vertices where either *κ*_*i*_ ≈ 0 or *τ*_*i*_ ≈ 0. These are the likely locations where a small change in the shape of the backbone can change its local topology, with a potentially substantial change in the protein’s biological function.

From Fig 2b) one observes that inflection points *i.e*. small *κ*_*i*_ values are extremely rare in crystallographic protein structures. Indeed, a generic space curve does not have any inflection points. At the same time general arguments state that generically at least one flattening point can be expected to be present. As shown in Fig 2b) flattening points where *τ*_*i*_ ≈ 0 do appear even though they are relatively rare in protein structures. Moreover, it is observed from the Figure that at a flattening point the bond angle values are mostly either *κ*_*i*_ ≈ 1 or *κ*_*i*_ ≈ *π*/2.

Since the torsion angles are defined *mod*(2*π*), in the case of discrete Frenet frames there is an additional structure: There is the line *τ*_*i*_ = ±*π* in Fig 2b) where the torsion angle has a 2*π* discontinuity, hence it can change sign by crossing the line. This multivaluedness is absent in regular space curves, with *τ*(*s*) a single-valued continuous function. But the limits *τ*_*i*_ → ±*π* can be thought of as the small curvature and large torsion limits of the Eq (7). Notably, *κ*_*i*_ = 1 and *τ*_*i*_ → ±*π* corresponds to an ideal, straight *β*-strand. Therefore a point on the line *τ*_*i*_ → ±*π* (or in its immediate vicinity) will be called a *β*-flattening point in the sequel.

**Fig 3:**
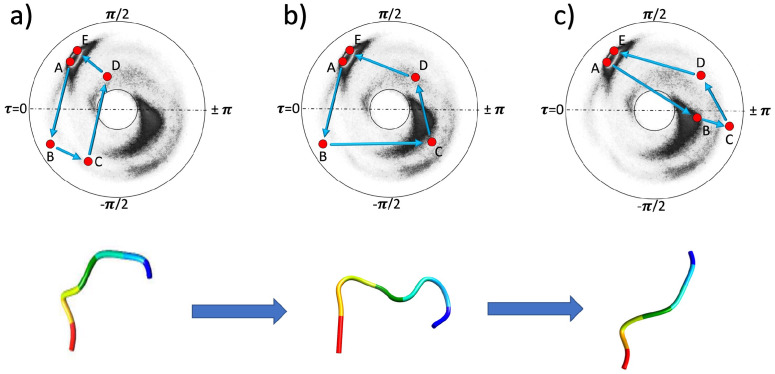
Different local topologies for a chain with five vertices. a) With two crossings of the flattening point line *τ* = 0. b) With one crossing of the flattening point line *τ* = 0 and one crossing of the line of *β*-flattening points *τ* = ±*π*. c) With two crossings of the line of *β*-flattening points *τ* = ±*π*. In both a) and c) the folding index Eq (17) vanishes but in b) the folding index has value -1 since the trajectory encircles the disk center once in counterclockwise direction. The attached 3D figures show the corresponding structures, ordered according to A = blue and E = red.

The multi-valuedness of the torsion angle *τ*_*i*_ affects the local topological invariants, in the case of a discrete chain. This is exemplified in the Fig 3. These Figures depict the three characteristic examples of a protein loop topology, when the loop interpolates between two right-handed *α*-helices; similar considerations apply when the interpolation is between any two generic points.

For clarity: Whenever a Figure similar to those in Fig 3 is drawn in the sequel, the two neighboring vertices *i.e*. values (*κ*_*i*_, *τ*_*i*_) and (*κ*_*i*+1_, *τ*_*i*+1_) are always to be connected by a virtual segment that is a straight line on the disk. Even though in reality those values of (*κ*, *τ*) that are on the straight line may not correspond to any actual atomic position along the piecewise linear C*α* backbone. At the level of local topology, this has no effect.

In all Fig 3, for convenience the initial points (A) and the final points (E) are fixed and located near the *α*-helical region where the torsion angle has a positive value close to *τ* ≈ 1 but this can be changed with no effect. At the points (B) and (C) the torsion angles are always negative in the Fig 3. At the point (D) the torsion angle is positive.

In the Fig 3a) the chain proceeds from point (A) to point (B) by crossing the line of flattening points *τ* = 0. It then continues to point (C). From there it proceeds and crosses the line of flattening points a second time, to arrive at point (D) where the torsion angle returns to a positive value. The chain then continues and ends at point (E). In this case the local topology is fully analogous to that of a regular curve. In particular, the two flattening points along the chain can be removed by a bi-flattening perestroika that lifts both points (B) and (C) above the line *τ* = 0 to positive *τ*_*i*_ values, without the chain crossing the inflection point *κ* = 0 at the center of the disk.

In the Fig 3b) the chain proceeds to the point (B), again by crossing the line of flattening points *τ* = 0. From there it proceeds to point (C) that is located near the *β*-stranded region. The chain then crosses the line of *β*-flattening points *τ* = ±*π* as it proceeds to point (D) where the torsion angle is positive. The chain finally ends at point (E) in the *α*-helical region. In this case there is only one flattening point along the chain, since the second time the torsion angle changes its sign at a *β*-flattening point. The chain encircles the inflection point once in the counterclockwise direction. Neither the flattening point nor the *β*-flattening point can be removed without encountering an inflection point perestroika along the chain.

The Fig 3b) motivates to introduce a winding number termed folding index *Ind*_*f*_ [39] for a backbone chain segment between sites *n*_1_ and *n*_2_. The folding index classifies loop structures and entire folded proteins by counting the number of times the chain encircles the inflection point. Its value can be obtained from the Eq (17)

$$\begin{array}{c} Ind_{f}=[\frac{\Gamma }{2\pi }]\;\;\;\text{with}\;\;\;\Gamma =\sum_{i=n_{1}+2}^{n_{2}-2}\{\begin{array}{cc} \tau_{i+1}-\tau_{i}-2\pi & \text{if}\;\tau_{i+1}-\tau_{i}>\pi \\ \tau_{i+1}-\tau_{i}+2\pi & \text{if}\;\tau_{i+1}-\tau_{i}<-\pi \\ \tau_{i+1}-\tau_{i} & \;\;\;\;\text{otherwise} \end{array} \end{array}$$ (17)

with [*x*] the integer part of *x*. Here Γ is the total rotation angle (in radians) that the chain makes around the inflection point in Figures such as Fig 3. The folding index is positive when the rotation is clockwise, and negative when the rotation is counterclockwise. In the Fig 3b) the folding index has the value *Ind*_*f*_ = −1 since the chain encircles the inflection point once in counterclockwise direction.

In the Fig 3c) the torsion angle changes its sign between points (A) and (B) and between points (C) and (D). Now the sign change occurs at *β*-flattening points. The general rules of local topology for the sign change at a *β*-flattening point are like those at a flattening point. In particular, a pair of *β*-flattening points can either be created or removed in a bifurcation called *β*-flattening perestroika that is akin a bi-flattening perestroika. In the Fig 3c) a *β*-flattening perestroika occurs if the vertices (B) and (C) are both moved upwards across *τ* = ±*π* line without the chain crossing the inflection point *κ* = 0 at the center of the disk.

The folding index is a local topological invariant. For example, in Fig 3b) its value does not change unless the chain is deformed so that either the flattening point between (A) and (B) or the *β*-flattening point between (C) and (D) becomes removed by an inflection point perestroika, that either converts the *β*-flattening point into a flattening point by a deformation that takes the chain in Fig 3b) into the chain in Fig 3a) or converts the flattening point into a *β*-flattening point by a deformation that takes the chain in Fig 3b) into the chain in Fig 3c). In both cases the final folding index vanishes.

The three examples in Fig 3 summarize all essential aspects of local topology that are encountered in the case of a discrete chain. In particular, the line of flattening points and the line of *β*-flattening points have a very similar character in terms of local topology. They can be interchanged by an inflection point perestroika that also changes the folding index.

For a comparison between the present description and the Ramachandran map, see [40].

## Results

The SARS-CoV-2 spike protein is a timely example of a protein that continues to evolve structurally, with significant biological (epidemiological) consequences. Moreover, the spike protein site 614 of the notorious D→G substitution is a good example of a site that is proximal to a flattening point. This motivates to select the flattening points of the spike protein as a starting point for presenting the general methodology. However, the methodology is quite independent of this choice, it is applicable to any kind of local topology changing bifurcation and to any protein backbone. As an example, it is shown that the recently observed N501Y substitution in the spike protein can provide an example of an inflection point bifurcation.

**Fig 4:**
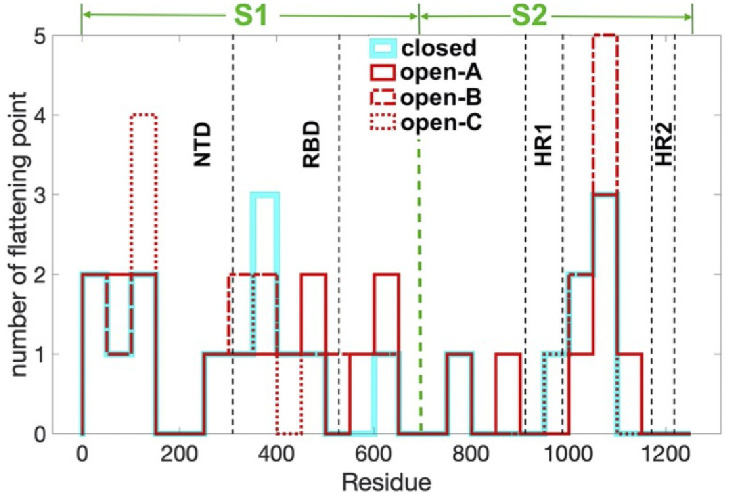
The histogram shows the distribution of sites that are proximal to a flattening point along the spike protein backbone, in blocks of 40 sites. The proximal sites accumulate in the NTD and RBD domains, and in the fusion core between the HR1 and HR2 domains. The PDB structures are 6VXX for closed state and 6VYB for open state.

First, the sites along the SARS-CoV-2 spike protein C*α* backbone that are proximal to a flattening point are classified. Their local neighborhoods are then inspected by comparisons with Fig 3, to investigate the potential bifurcations. The Table 1 lists all those SARS-CoV-2 spike protein C*α*-sites that are proximal to a flattening point in the PDB structures 6VXX (closed state) and 6VYB (open state). Here a torsion angle value is determined to be proximal to a flattening point when |*τ*_*i*_| < 0.2; other (small) values could also be considered. This value is chosen since in terms of distance, it is less than the radius of a carbon atom: With 3.2 Å the diagonal length of a peptide plane, a distance of 0.2 radians in angle between two points corresponds to a distance slightly above 0.6 Å in length. The histogram in Fig 4 shows the distribution of the flattening point sites in relation to spike protein backbone. There are relatively many entries in the NTD and RBD domains, and in the fusion core between the HR1 and HR2 domains. Notably the number of proximal sites is also different in the closed and open states of the spike protein, there are more proximal sites in the open state. Thus a transition between the two states involves changes in local topology that affect in particular the NTD and RBD domains, and the HR1-HR2 junction.

**Table 1:** Flattening points in spike protein PDB structures 6VXX and 6VYB. Columns are organized as follows: Residue number with present amino acid. CL stands for cluster with smallest RMSD (in Å); clusters are taken from [41]. MC stands for most common mutagenic amino acid substitutions in the cluster. P stands for preceding, A stands for adjacent and F stands for following fragment. Closed (c) and open (o) denotes the state where flattening point is observed. A,B,C denotes the monomer of spike protein.

Residue	CL/P	RMSD/P	MC/P	CL/A	RMSD/A	MC/A	CL/F	RMSD/F	MC/F	closed/open
40D	24	0.69	A	I	0.69	V/S/N	X	0.58	L/A/S/T	c / o
46S	24	0.92	A	16	0.82	G	10	0.63	G	c / o
59F	7	0.44	G	26	0.18	G	XI	0.24	G	c / o
98S	XII	0.84	A	14	0.78	V	X	0.3	A/T	o-A
103G	9	0.77	I/T	VI	0.21	A	25	0.38	A/V/T	c / o-A
109T	II	0.74	G	6	0.54	G	X	0.55	A/L	c / o
114T	26	0.87	A	XI	0.71	A/G/S	12	0.35	S/V	o-C
124T	17	0.74	G	III	0.5	G	21	0.5	G	o-BC
138D	XII	0.39	S/A	6	0.39	G	25/X	0.72	A/V	o-C
287D	24	0.17	A	VI	0.18	A/G	25	0.23	T/L/A/V	c / o
316S	24	0.21	A	VI	0.45	A/G	25	0.41	T/L/A/V	o
324E	24	0.27	A	I	0.39	A	25	0.34	T/L/A/V	c / o-B
376T	VII	0.83	A/G	I	0.36	A	25	0.34	A/V	c / o-BC
382V	26	1.18	A/T	IX	0.72	A/T	25	0.86	A/T	c
394N	24	0.73	A	I	0.36	A	25	0.13	T/A	c / o
422N	IX	1.06	G	IX	1.13	T	20	0.87	E/V/D	c
423Y	IX	1.13	G	20	0.87	L/H	25	0.27	T/A/L/V	o-AB
459S	XII	1.10	A	14	0.58	V	12	0.82	V	o-A
464F	17	0.21	G	26	0.13	G	XI	0.23	G	c / o
545G	17	0.69	G	26	0.51	G	XI	0.6	G	o-B
565F	V	1.15	S/T/D/E	VI	0.21	A/G	25	0.28	A/T/V/L	o-C
575A	24	0.22	A	VI	0.19	A/G	25	0.33	A/T/V	o-AB
605S	IX	0.83	G	20	1.0	L	V	1.14	L/A/T/V	o
614D	XII	0.76	S/A/N/G	26	0.75	G	10	0.42	G	c / o-A
758S	V	0.76	T	13	0.94	S	IV	0.61	L	c / o
892A	X	0.71	V	VIII	1.145	V	25/12	0.73	V	o-A
974S	19	0.74	H	I	0.78	A	V	0.59	A/V/L	c / o-C
1035G	IX	0.93	G	IX	0.51	T	12	0.62	S/V	c / o-BC
1041D	VII	0.67	A/T/G	26	0.54	G	IV	0.67	L/N	c / o
1058H	17	0.64	G/A/S	III	0.64	G	21	0.52	G	o-B
1080A	24	0.87	A	I	0.26	A	25	0.26	A/T/V	c / o
1084D	24	0.78	A/S	14	1.33	N/V/S	III	0.3	N	c / o
1092E	24	0.48	A	14	0.87	V/S	21	0.56	G	c / o
1093G	14	0.87	L	21	0.56	V	28	0.76	A/V/L	o-B
1131G	VII	0.53	A/G/T	6	0.43	G	XI	0.39	G	o-AB

In the case of a bond angle, here the value is considered small when *κ*_*i*_ < 0.5. General arguments state that inflection points are not generic, and Fig 2b) shows that there are indeed very few sites that are close to an inflection point. For the spike protein the smallest value is *κ*_*i*_ = 0.39 and it is located at the site 103 that also appears in Table 1.

The local geometry of all the individual residues that are proximal to a flattening point has been investigated and the potential for mutations to have large effects for residues proximal to flattening points has been estimated, by comparison to Fig 3. For this, a combination of statistical analysis and stereochemical constraints has been utilized. The Table 1 also summarizes these findings.

The statistical analysis employs the classification scheme of Protein Data Bank structures described in [41]. This scheme decomposes a C*α* backbone into fragments that consist of backbone segments with six successive sites; in accordance with Eqs (11) and (12) a fragment with six sites determines three pairs of bond and torsion angles. In [41] the fragments that appear in high resolution PDB structures have been organized into disjoint clusters. To assign a cluster to a fragment, there must be at least one other fragment in the same cluster within a prescribed RMS cut-off distance; in [41] the cut-off is 0.2 Å. Two clusters are then disjoint, when the RMSD between any fragment in the first cluster and any fragment in the second cluster exceeds this RMS cut-off distance. It was found that around 38% of protein loops in the high resolution PDB structures can be decomposed into fragments that belong to twelve disjoint clusters, labeled I-XII in [41]. When fragments from an additional set of 30 disjoint clusters are included, the coverage increases to ∼ 52% [41].

In the Table 1 both cluster sets I-XII and 1-30 appear; the notation of [41] is used throughout in the following. Beyond these two sets, the clusters become increasingly smaller, and in the present study those smaller clusters have not been considered. The somewhat low resolution of the available spike protein structures in comparison to the very high resolution structures used in [41] does not justify a more detailed scrutiny.

The clusters in the Table 1 have been identified as follows: A pair of bond and torsion angles (*κ*_*i*_, *τ*_*i*_) at the *i*^*th*^ site of the spike protein that is a proximal site to a flattening point can be assigned to three different clusters. The first cluster describes the bond and torsion angles for the sites (*i* − 2, *i* − 1, *i*); this cluster is labelled P (for Preceding) in the Table 1. The second cluster that is labelled A (for Adjacent) in the Table 1 describes the angles for sites (*i* − 1, *i*, *i* + 1). The third cluster is labelled F (for Following) and it describes the angles at spike protein sites (*i*, *i* + 1, *i* + 2). The cluster that provides the best match to the spike protein is listed in the Table 1 and it is determined as follows: Let (*x*_*a*_, *y*_*a*_, *z*_*a*_) denote the six space coordinates of a segment that corresponds to three consecutive pairs of (*κ*, *τ*) values in the spike protein. Let (*x*_*k*,*a*_, *y*_*k*,*a*_, *z*_*k*,*a*_) be the corresponding six space coordinates of a *k*^*th*^ fragment in each of the clusters of [41]. The best matching cluster in Table 1 is the one that contains a fragment with the minimal root-mean-square distance (RMSD) to the given spike protein segment (in units of Ångström):

$$\begin{array}{c} \Delta_{min}\;=\;\underset{clusters}{\text{min}}\sqrt{\underset{k}{\text{min}}\sum_{a=1}^{6}{\left(x_{a}-x_{k,a}\right)}^{2}+{\left(y_{a}-y_{k,a}\right)}^{2}+{\left(z_{a}-z_{k,a}\right)}^{2}} \end{array}$$ (18)

where min stands for minimization. Once the best matching cluster is determined, the corresponding statistical distribution of amino acids found in [41] is used to identify those amino acids that are most probable to appear at the *i*^*th*^ site of the spike protein, in case of a substitution. If the size of this statistically most probable amino acid is smaller than the size of the present amino acid at the *i*^*th*^ site, a substitution is considered to be sterically possible without a wider rearrangement of the backbone conformation so that a bifurcation is likely to take place in its vicinity. But if the size is larger, a substitution likely requires a wider extended rearrangement of the spike protein conformation. This can be energetically costly and entail a relocation of the bifurcation site. Thus, one can expect that a smaller residue should in most cases be more likely to be better tolerated than a larger residue. In fact a larger residue may or may not be tolerated at all, depending on the structural context. Therefore, as a reasonable approximation smaller residues are here considered to be the most probable substitutions.

The Table 1 lists the most probable substitutions that are predicted in this manner.

### Example: The D614G mutation of spike protein

**Fig 5:**
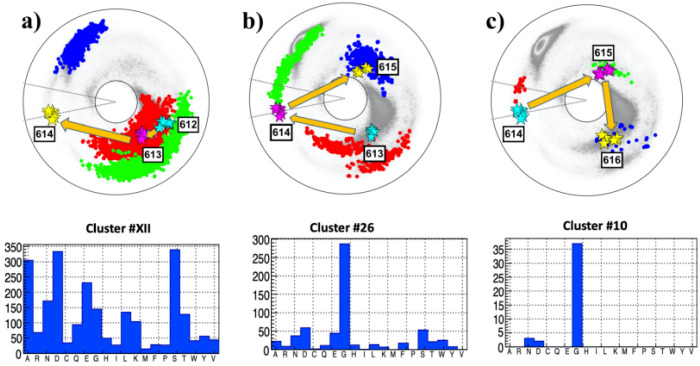
The (*κ*_*i*_, *τ*_*i*_) spectrum for *i* = 612 − 616; this engages sites 610 − 617 along the C*α* backbone. a) The spectrum for preceding sites P:(612, 613, 614), in the background of the best matching cluster #XII in [41]. The most common amino acids in the cluster are S, D, A, E, N, G. b) The spectrum for adjacent sites A:(613, 614, 615), in the background of the best matching cluster #26 in [41]. The most common amino acid in the cluster is G. c) The spectrum for following sites F:(614, 615, 616), in the background of the best matching cluster #10 in [41]. The most common amino acid in the cluster is G.

The Table 1 identifies the site 614 of the spike protein, where the D→G substitution has occurred, as a site that is proximal to a flattening point. Thus, this site is chosen to exemplify the present methodology. The local topology and its potential bifurcations is analyzed using figures such as Fig 5. Each of the three Figures depicts the three bond and torsion angle pairs for the three backbone segments (P, A and F respectively) that include the angles of the site 614.

The legend is as follows: In figures such as Fig 5, the vertices that mark the C*α* sites of spike protein are always identified by stars. These stars are color-coded and organized according to increasing site number following the Cyan-Magenta-Yellow (CMY) color table. The dotted background comprises the C*α* sites of all the fragments in the corresponding best matching cluster. The background sites are always ordered similarly using the Red-Green-Blue (RGB) color table. The adjacent histogram displays the amino acid distribution in the best matching cluster, using the data that is obtained from [41].

The Fig 5a) shows the bond and torsion angles for the sites P:(612,613,614) of the spike protein. The best matching preceding (P) cluster is also shown, it is the cluster #XII of [41]. From Table 1 the RMSD (18) between the spike protein segment and the cluster has the value Δ_*min*_ ≈ 0.76Å. This is clearly larger than the 0.2 Å cut-off used in [41]. Nevertheless, the cluster is included in the analysis since the resolution at which the spike protein PDB structures (6VXX, 6VYB and 6XS6) have been measured is also clearly smaller than the resolution of the structures used in [41]. This justifies that despite the relatively large value of Δ_*min*_ the present analysis proceeds with cluster #XII, keeping in mind that the Δ_*min*_ is not very small.

The statistical analysis [41] shown in Fig 5a) proposes that the most probable substitution at site 614D are to S, A, N and G; the amino acid E s excluded in the present study on sterical grounds since it has a larger size than D.

The best matching cluster for the adjacent sites A:(613,614,615) shown in Fig 5b) is the cluster #26 of [41], with Δ_*min*_ ≈ 0.75Å which is again relatively large. The statistical analysis [41] now proposes that the most probable substitution at site 614 is D→G; the probability for any other amino acid substitution is very low. In particular the probability for D itself is low, suggesting instability.

Finally, the best matching cluster for the following sites F:(614,615,616) shown in Fig 5c) is #10. The RMSD has now a somewhat lower value Δ_*min*_ ≈ 0.41Å. The statistical analysis [41] proposes that the most probable substitution at site 614 is again D→G. The probability for any other substitution is very low. In particular the probability for D itself is low, again suggesting instability of the residue.

The combined spike protein chain shown in the three Fig 5, starting from site 612 in Fig 5a) and ending at 616 in Fig 5c), encircles the inflection point once in clockwise direction. Thus the folding index has value +1 and, except for the direction and the location of the end points, the topology of the trajectory is similar to that in Fig 3b). In particular, both a flattening point and a *β*-flattening point occur at neighboring segments along the chain. This proposes that the site 614 is a potential bifurcation hot-spot, prone to a change in the local topology by an inflection point bifurcation. The bifurcation can change the local topology from that resembling Fig 3b) to one that resembles either Fig 3a) with a bi-flattening point or Fig 3c) with a pair of *β*-flattening points. Since G is the only amino acid that consistently appears in all three clusters and since there is no obvious steric hindrance for a D→G substitution, the prediction of the present analysis is that a D→G substitution is probable at the site 614 of spike protein; this is the notorious D614G mutation that has already been observed.

A comparison of the three PDB structures 6VXX, 6VYB with 6XS6 shows that apparently the mutation has not caused any change in local topology, at least according to available structures using the available resolution.

### Further examples of flattening point hot-spots in spike protein

The Table 1 proposes that there is quite a large number of potential bifurcation hot-spots in the spike protein that can be similar to 614D, with a proximal flattening point. In the sequel a selection of these sites is analyzed. The three examples analyzed here, taken from the NTD domain with site numbers 59, 103 and 287, are representative but not necessarily the most probable hot-spot sites; the example at the site 103 is included since this is the site with the lowest bond angle value along the entire spike protein backbone. An example from the fusion core between HR1 and HR2 domains with site number 1080 is also presented. Finally, the local topology around the site 501 where the N→Y substitution has recently been observed [32] is also analyzed and its bifurcation potential is investigated.

#### The residue 59F

**Fig 6:**
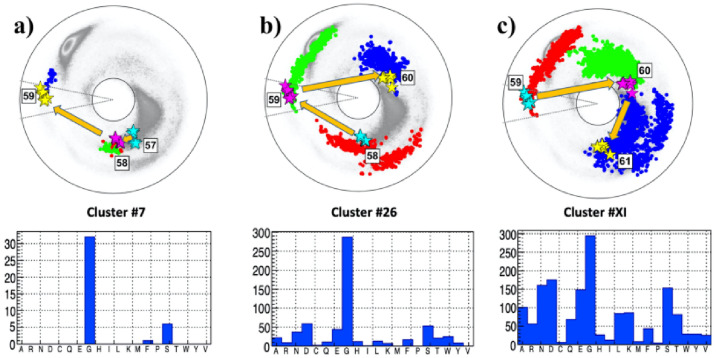
The (*κ*_*i*_, *τ*_*i*_) spectrum for *i* = 57 − 61; this engages sites 55 − 62 along the C*α* backbone. a) The spectrum for P:(57, 58, 59), in the background of the best matching cluster #7 in [41]. b) The spectrum for A:(58, 59, 60), in the background of the best matching cluster #26 in [41]. c) The spectrum for F:(59, 60, 61), in the background of the best matching cluster #XI in [41]. The most common amino acid in all three clusters is G.

The Fig 6 show the neighborhood of the site 59F, located in the NTD domain of subunit S1. The Figures reveal that the topology of the trajectory from site 57 to 61 is very similar to that in the case of site 614, shown in Fig 5: There is a residue that is proximal to a flattening point and right after it there is a residue that is proximal to a *β*-flattening point. The folding index has value +1 since the trajectory encircles the inflection point in clockwise direction. Thus, as in the case of 614, the site 59 is prone to an inflection point bifurcation such as those described in Fig 3. The RMSD values (18) are all quite small, indeed clearly smaller than in the case of 614D, so that the three clusters that are identified in the Fig 6 are a very good match. The statistical analysis of all three clusters show that G has a very high probability at the site 59; both S and D have some propensity albeit much smaller than G while the probability of the existing amino acid F is very small. Thus the site 59 is a very good candidate for a F→G substitution.

#### The case of 103

**Fig 7:**
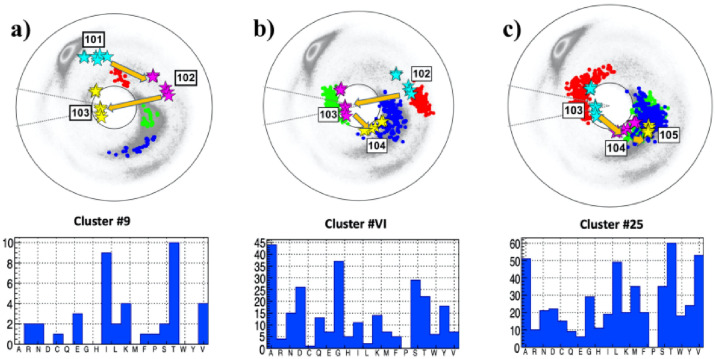
The (*κ*_*i*_, *τ*_*i*_) spectrum for *i* = 101 − 105; this engages sites 99 − 106 along the C*α* backbone. Panel a) The spectrum for P:(101, 102, 103), in the background of the best matching cluster #7 in [41]. The most common amino acids in the cluster is T, followed by I. Panel b) The spectrum for A:(102, 103, 104), in the background of the best matching cluster #26 in [41]. The most common amino acid in the cluster is A, followed by G. Panel c) The spectrum for F:(103, 104, 105), in the background of the best matching cluster #XI in [41]. The most common amino acid in the cluster are T and L, followed by A and V.

The Fig 7 show the neighborhood of the site 103G, located in the NTD domain of subunit S1. Here the situation is somewhat exceptional, since the residue 103 is already the smallest amino acid G.

The chain between sites 101 and 105 is shown in Fig 7. It has one vertex near a *β*-flattening point at 102. This is immediately followed by the vertex 103 that is proximal both to a flattening point and to the inflection point. The following vertex 104 has also a very small bond angle value. The overall shape of the trajectory suggests a bifurcation hot-spot with inflection point perestroika that converts the site 103 from a vertex that is proximal to the flattening point into a vertex that is proximal to a *β*-flattening point. It is also plausible that there has been a recent substitution with ensuing inflection point perestroika, that has converted the local topology by moving the vertex 103 from the vicinity of a *β*-flattening point to the vicinity of a flattening point.

The statistical analysis shows that A, which is the smallest amino acid after G, has the highest propensity in the case of the adjacent cluster, shown in Fig 7b). The propensity of A is also larger than that of G in the following cluster shown in Fig 7c). Both clusters have also small RMSD value. On the other hand, in the histogram of the preceding cluster shown in Fig 7a) the amino acid A is absent. However, RMSD value is not very small, and the Figure also shows that cluster #9 can not be good match to the spike protein segment P:(101,102,103): The distance between the observed *τ* value at the site 103 deviates from the *τ*-values in the cluster by some 150 degrees. Thus the conclusion is that the cluster #9 should be used with care, for a bifurcation outcome prediction.

**Fig 8:**
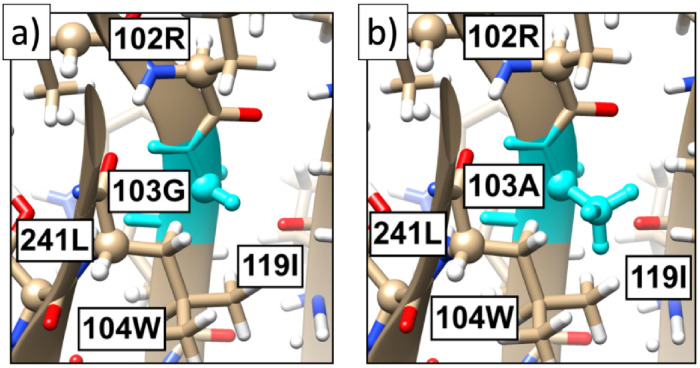
a) The present all-atom structure of the spike protein in the neighborhood of site 103. b) The substitution of A in place of 103G, as predicted by Chimera [42]. There are no apparent steric hinders for the substitution.

The Fig 8a) shows the present 3D spike protein structure in the neighborhood of the site 103. In the Fig 8b) the amino acid G has been replaced by A; the effect of this substitution is estimated using a crude energetic analysis with Chimera [42]. An inspection of the interatomic distances show that A can be substituted for G without encountering steric clashes. But in the case of the other amino acids T, V and L that also have a high propensity in the cluster of Fig 7c), a substitution using Chimera leads to steric clashes. This proposes that a substitution is accompanied with a wider conformational rearrangement.

The conclusion of the present analysis is that the site 103G is a potential G→A bifurcation hot-spot.

#### The case of 287D

**Fig 9:**
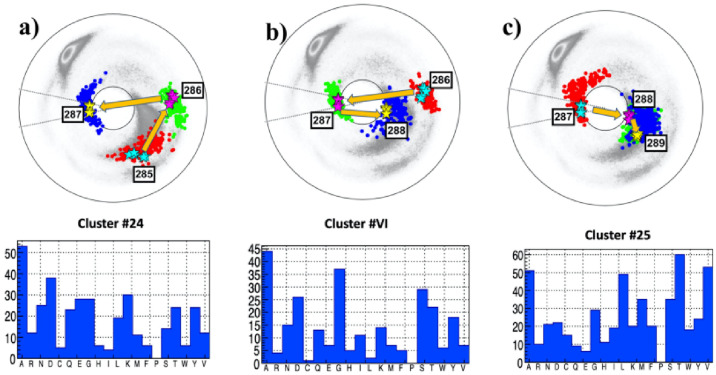
The (*κ*_*i*_, *τ*_*i*_) spectrum for *i* = 285 − 289; this engages sites 283 − 290 along the C*α* backbone. Panel a) The spectrum for P:(285, 286, 287), in the background of the best matching cluster #24 in [41]. The most common amino acids in the cluster is A, followed by D. Panel b) The spectrum for A:(286, 287, 288), in the background of the best matching cluster #VI in [41]. The most common amino acid in the cluster is A, followed by G. Panel c) The spectrum for F:(287, 288, 289), in the background of the best matching cluster #25 in [41]. The most common amino acid in the cluster are T and V, followed by A and L.

The Fig 9 show the neighborhood of the site 287D, located in the NTD domain of subunit S1. The site is both preceded and followed by a site that is proximal to a *β*-flattening point. The folding index has value -1 since the chain encircles the inflection point in counterclockwise direction. But since 286 and 288 are both very close to a *β*-flattening point the chain passes back-and-forth very close to the inflection point.: The links 286-287 and 287-288 have a very similar topology to the links 102-103 and 103-104 in Fig 7.

The RMSD values (18) are all very small so that the three clusters identified in the Fig 9 are an excellent match. The statistical analysis in all three clusters show that A has a very high probability at the site 287; both T and V are also likely substitutions in the following cluster, and G has also propensity in the adjacent cluster. But D that is now located at the site, is not very prominent in any of the clusters. Thus the site 287 is a very good candidate for a D→A bifurcation hot-spot.

### The local topology of the N501Y mutation site

Thus far, in the present article, only those bifurcation hot-spot sites that are in the vicinity of a flattening point have been analyzed. However, the three local topologies in Fig 3 can give rise to various other kind of bifurcations as well. For this, the local topology of the recently observed new mutation at site 501N with N→Y substitution [32] is now analyzed, for its bifurcation potential.

**Fig 10:**
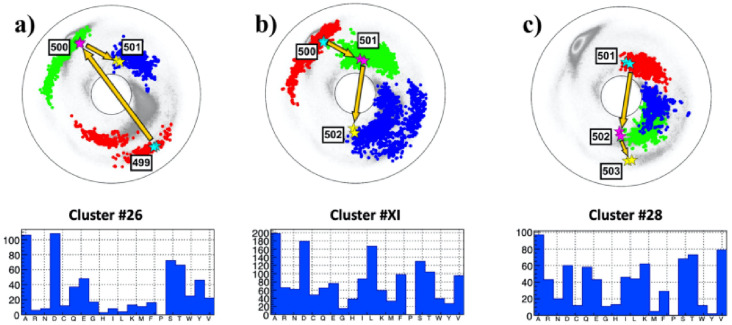
The (*κ*_*i*_, *τ*_*i*_) spectrum for *i* = 499 − 503; this engages sites 497 − 504 along the C*α* backbone. Data for 501N is only available in the open state chains B and C. Panel a) The spectrum for P:(499, 500, 501), in the background of the best matching cluster #26 in [41]. The most common amino acids in the cluster are A and D. Notably, Y is more common than N. Panel b) The spectrum for A:(500, 501, 502), in the background of the best matching cluster #XI in [41]. The most common amino acid in the cluster are A and D; now N is more common than Y. Panel c) The spectrum for F:(501, 502, 503), in the background of the best matching cluster #28 in [41]. The most common amino acid in the cluster is A, there is also propensity to D but V is more common, N appears but Y is absent.

The Fig 10 describe the local topology of the site 501 prior to the N→Y substitution; data is presently available only for the chains B and C in the open state of 501N. In particular, no structural data is available for the mutated 501Y at this time. Except for the direction and the location of the end points, the topology of the trajectory in Fig 10 is akin that in Fig 3c) with two crossings of the line of *β*-flattening point (but very near to the inflection point). There is no site in the vicinity of *τ* = 0 in Fig 10. Thus a bifurcation that relates to a flattening point, similar to that in the case of the D614G, appears unlikely.

Notably both the segment connecting 499 to 500, and the segment connecting 501 to 502 pass very close to the inflection point *κ* = 0. This suggests that the local topology can be prone to a variant of an inflection point bifurcation that causes a transition either into the topology akin that in Fig 3a) or that in Fig 3b).

**Fig 11:**
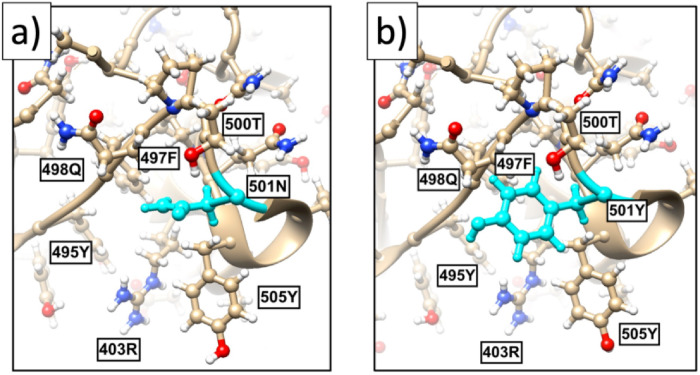
a) The present all-atom structure of the spike protein in the neighborhood of site 501. b) The effect of N→Y substitution as predicted by Chimera [42]. Besides a small change in the orientation of 505Y, despite the substantially larger size of Y in comparison to N there is no apparent steric hinder for the N→Y substitution at site 501.

In Fig 10a) and 10b) the RMSD values (18) are very small; in the case of Fig 10a) the value is Δ_*min*_ = 0.2 and in the case of Fig 10) the value is Δ_*min*_ = 0.16. Thus both spike protein segments are well represented by the ensuing clusters. But in the case of Fig 10c) the value is much larger, Δ_*min*_ = 0.7 so that this segment is not well represented by the cluster. According to all three histograms in Fig 10 the likelihood of the observed N→Y substitution at site 501 should be lower than substitutions N→A or N→D by the similarly hydrophobic A and D. Furthermore, since Y has a much larger size than N, a priori the N→Y substitution requires an extensive conformational rearrangement which can be energetically costly. But a crude energetic Chimera [42] analysis of the neighborhood around the site 501N that is summarized in Fig 11 reveals that there is a “pocket” inside the spike protein structure that is large enough to accommodate Y with no major conformational re-arrangement; only a change in the orientation of 505Y is observed. Since the N501Y substitution is not necessarily in contrast with stereochemical considerations, the energetic cost of a substitution can be minor.

The likely bifurcation that can accompany the N→Y substitution can be deduced by an analysis of all those fragments in the (Preceding) cluster #26 of Fig 10a) where Y is in the third (Blue) position; this is the cluster where Y has the highest prevalence. The (Adjacent) cluster #XI can be analyzed similarly. But in the (Following) cluster #28 where the spike protein segment has a relatively low quality match, there is no Y.

**Fig 12:**
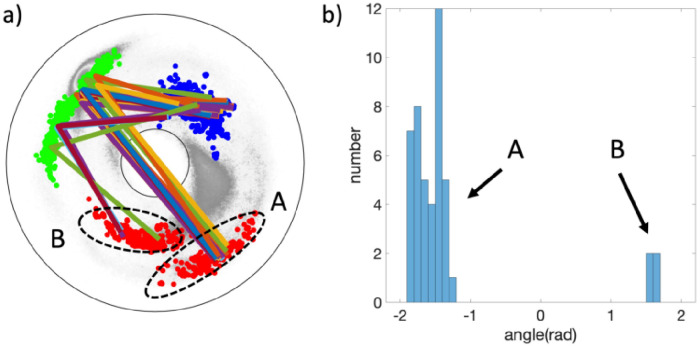
a) The fragments of cluster #26 where Y appears in the last (Blue) site can be divided into two disjoint subsets, labeled A and B. b) The subsets A and B differ by a ∼180 degree rotation (flip) of the peptide plane between the second (Green) and third (Blue) site, around the connecting Frenet frame tangent vector **t**.

This analysis starts with Fig 12a): The initial (Red) dots of the fragments in the cluster #26 are naturally divided into two disjoint subsets. There are 42 backbone fragments that start in the subset labeled A in the Fig 12, and there are 4 fragments that start in the subset labeled B, with Y in the last (Blue) position in all fragments. The fragments in the subset A all pass very close the inflection point *κ* = 0, in a manner which is very similar to the 499-501 segment in Fig 10. The fragments in the subset B all cross the *τ* = 0 line in a manner that is topologically similar to the A-B-C segments in Fig 3a) and 3b) (except for orientation). A comparison of the two subsets A and B in the Fig 12b) reveals that there is a transition akin the peptide plane flip observed in [43]. For this, define the normal vector of the peptide plane between the C*α*(*i* − *i*) and C*α*(*i*), as the cross product between the vector **t**_*i*_ and the vector that points from C*α*(*i* − *i*) to the O(*i*) atom of the corresponding peptide plane. Similarly, define the normal vector of the peptide plane between C*α*(*i*) and C*α*(*i* + 1). Then, evaluate the angle between these two normal vectors. The result is shown in the histogram of Fig 12b): For all entries in the subset A of Fig 12a) the angle has a value which is very close to −*π*/2 while for all entries in the subset B the angle has a value that is very close to + *π*/2. The sign is determined by comparison to the virtual plane that is defined by C*α*(*i* − 1), C*α*(*i*) and C*α*(*i* + 1).

The present analysis proposes that the peptide plane between sites 500 and 501 of spike protein can be prone to an inflection point bifurcation where the peptide plane flips, corresponding to a rotation of ∼180 degrees around the Frenet frame vector **t** between the ensuing C*α* atoms.

**Fig 13:**
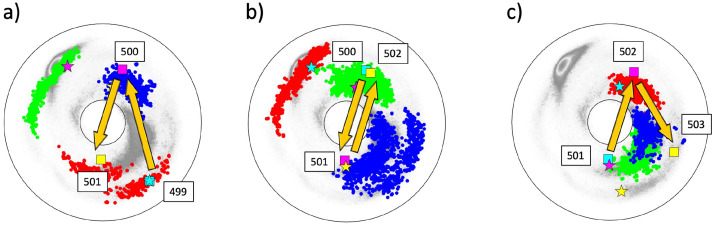
A hypothetical trajectory that rotates the direction of the C*β* atom at site 501 so that it turns and points to the opposite direction. The starred vertices are the same as in Fig 10, the vertices denoted by squares correspond to the hypothetical trajectory; the labeling a), b), c) is in direct correspondence with Fig 10.

**Fig 14:**
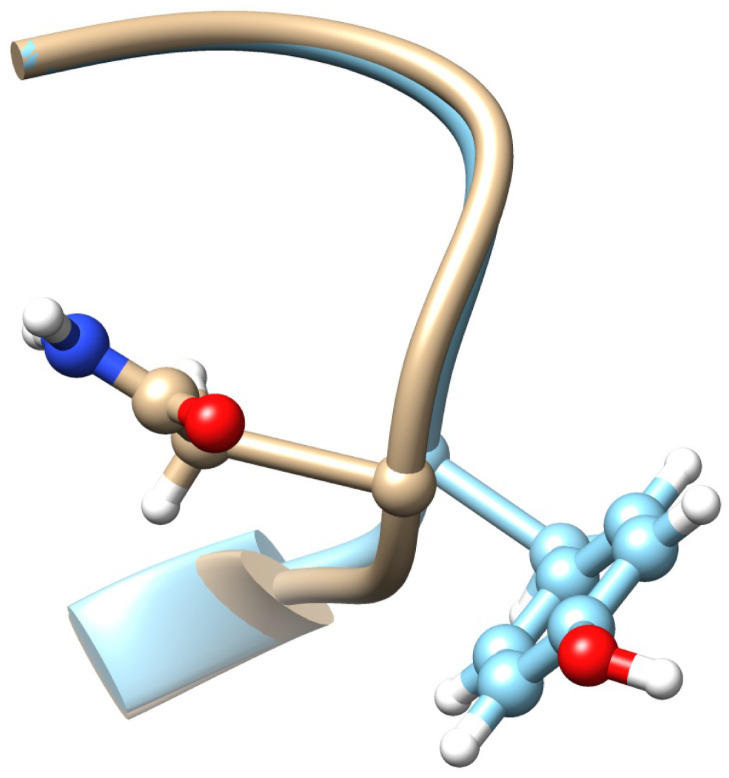
Chimera plot of the hypothetical 501Y fragment, with C*β* atom rotated to point in opposite direction from that of the C*β* at 501N. The deformation of the backbone is determined using the methodology described in [43, 44].

Finally, it is noted that the site 501 is on the surface of the spike protein where it can be exposed to solvent (water). But the N side chain is located in the “pocket” as shown in Fig 11, which is large enough to accommodate the Y side chain. It is plausible that when interaction with ACE2 occurs, it engages Y in such a manner that this side chain becomes rotated away from the “pocket”, for increased affinity with ACE2. The Fig 13 describe a *hypothetical* scenario how this can take place. In these Figures the C*α* backbone in the neighborhood of the site 501 has been deformed, so that the N→Y substitution takes place with the C*β* atom of the Y side chain pointing to the opposite direction of the observed C*β* atom at the side chain 501N. The interrelation between the direction of the C*β* side chain and the deformation of the C*α* backbone has been determined using the methods described in [43, 44]. The deformation of the C*α* backbone is local, it engages only the sites 499-503, as shown in Fig 14; beyond these sites the C*α* backbone coincides with the original spike protein backbone and there are no steric conflicts in the deformed backbone. Furthermore, as seen in Fig 11 there is no steric hinder, by the other amino acid side chains, that would prevent the Y side chain from rotating between the two positions: The Fig 14 shows the deformed backbone, after the Y side chain has been rotated away from the “pocket” so that it points directly to the solvent.

Comparison between the Figs 11 and 13 reveals that the transition between the two states, where the 501Y is embedded in the “pocket” and where it is exposed and points towards the solvent, engages a trajectory with four crossings of the line of *β*-flattening point. The two additional crossings, in comparison to Fig 11, are needed for the 180 degree rotation of the C*β* at site 501, so that it points to the opposite direction. Notably, the trajectory in this hypothetical example passes twice very close to the inflection point *κ* = 0. Alternative hypothetical trajectories can be considered. But local topology ensures that any alternative trajectory that is free from steric conflicts, shares the local topology of the one presented here, unless they differ by additional bifurcations.

## Conclusions

Topological techniques are commonly regarded to be among the most effective ones for addressing a wide range of physical problems. In particular in the case of a protein, where conformation is pivotal to biological function, topology should be a most valuable tool. However, thus far applications of topological methods in protein studies, and more generally in the study of filamental (bio)molecules such as DNA and RNA and other (bio)polymers, have been relatively sparse. In particular, the methods of local curve topology introduced and developed by Arnol’d have thus far not appeared at all, neither in a physical nor in a biological context.

The topological methodology that is developed here builds directly on Arnol’d mathematical work, and adapts it into the case of a discrete chain. The methodology is designed to investigate bifurcations that can change the local topology of discrete chain such as the protein backbone. The particular problem that is addressed here, is to try and pinpoint those hot-spot residues where a substitution can have the most profound conformational consequences: The hot-spot residues are those sites that are proximal to bifurcations where the local topology can change. The present methodology identifies these hot-spots using local topological considerations in combination with a comparative analysis that uses Protein Data Bank structures. But the methodology does not aim to actually predict, whether mutations at these hot-spot residues are more likely than at other sites. This needs to be deduced by other methods.

Since the underlying mathematical structure is relatively new, it was developed mainly by Arnol’d starting late 1990’s, and since it has thus far not found any (bio)physical applications, the present study serves as an invitation to apply these powerful methods of local topology to relate the structure and function of proteins, and to apply them to study the physical properties of biomolecules and filamental structures also more widely.

Here the methodology is developed using the spike protein of the SARS-CoV-2 virus as an example. Any other protein could be used equally but the choice of the spike protein is timely, reflecting the current pandemic situation. This choice also brings some downside, as the available structural data on the spike protein has been measured with relatively low resolution and is also partially lacking, at the time of writing: The Protein Data Bank structures that are used in the present study have been determined using Cryo-EM with a resolution no better than around 2.8–3.5 Å. The low resolution causes also some uncertainty in the proper identification of sites that are proximal to a flattening point. At the same time the statistical analysis that is used here for the identification of the pertinent PDB clusters is also preliminary, as it is based on a somewhat limited set of structural clusters that are measured with ultra high resolution. Therefore, a further development and refinement of the underlying statistical methodology is desired. Finally, the present article concerns only the local topology of a protein backbone. A more complete analysis should combine topological investigations with energetic studies. But that brings a practical limitation, as the presently available all-atom molecular force fields are still not very accurate and demand substantial computational resources. Here Chimera has been employed, simply because it is a method that can provide crude energetic estimates in the case complex proteins such as the spike protein, without undue need for computer resources.

## References

[1] Eguchi T, Gilkey PB, Hanson AJ. Gravitation, gauge theories and differential geometry. Physics reports. 1980;66(6):213–393. doi: 10.1016/0370-1573(80)90130-1

[2] Eschrig H. Topology and geometry for physics. vol. 822. Springer Science & Business Media; 2011.

[3] Nakahara M. Geometry, topology and physics. CRC Press; 2003.

[4] Strogatz S. H. Nonlinear dynamics and chaos with with applications to physics, biology, chemistry, and engineering CRC Press; 2014

[5] Flapan E, Wong H (Eds.) Topology and Geometry of Biopolymers AMS Contemporary Mathematics 2020;746

[6] Arnold V. I. (Ed.) (1994) Dynamical Systems V: Bifurcation Theory and Catastrophe Theory Encyclopaedia of Mathematical Sciences Vol. 5, Springer Verlag; 2014

[7] Arnold V. Singularities of caustics and wave fronts. vol. 62. Springer Science & Business Media; 2013.

[8] Arnold V. The geometry of spherical curves and the algebra of quaternions. Russian Mathematical Surveys. 1995;50(1):1.

[9] Arnold V. On the Number of Flattening Point on Space Curves. AMS Trans, Ser. 1996;171:11.

[10] Zhu N, Zhang D, Wang W, Li X, Yang B, Song J, et al. A novel coronavirus from patients with pneumonia in China, 2019. New England Journal of Medicine. 2020; 382:727–733 doi: 10.1056/NEJMoa2001017 PMC7092803 31978945

[11] Lu R, Zhao X, Li J, Niu P, Yang B, Wu H, et al. Genomic characterisation and epidemiology of 2019 novel coronavirus: implications for virus origins and receptor binding. The Lancet. 2020;395(10224):565–574. doi: 10.1016/S0140-6736(20)30251-8 32007145 PMC7159086

[12] Andersen KG, Rambaut A, Lipkin WI, Holmes EC, Garry RF. The proximal origin of SARS-CoV-2. Nature medicine. 2020;26(4):450–452. doi: 10.1038/s41591-020-0820-9 32284615 PMC7095063

[13] Benvenuto D, Giovanetti M, Ciccozzi A, Spoto S, Angeletti S, Ciccozzi M. The 2019-new coronavirus epidemic: evidence for virus evolution. Journal of medical virology. 2020;92(4):455–459. doi: 10.1002/jmv.25688 31994738 PMC7166400

[14] Paraskevis D, Kostaki EG, Magiorkinis G, Panayiotakopoulos G, Sourvinos G, Tsiodras S. Full-genome evolutionary analysis of the novel corona virus (2019-nCoV) rejects the hypothesis of emergence as a result of a recent recombination event. Infection, Genetics and Evolution. 2020;79:104212. doi: 10.1016/j.meegid.2020.104212 32004758 PMC7106301

[15] Riou J, Althaus CL. Pattern of early human-to-human transmission of Wuhan 2019 novel coronavirus (2019-nCoV), December 2019 to January 2020. Eurosurveillance. 2020;25(4):2000058. doi: 10.2807/1560-7917.ES.2020.25.4.2000058 PMC7001239 32019669

[16] Zhao S, Lin Q, Ran J, Musa SS, Yang G, Wang W, et al. Preliminary estimation of the basic reproduction number of novel coronavirus (2019-nCoV) in China, from 2019 to 2020: A data-driven analysis in the early phase of the outbreak. International journal of infectious diseases. 2020;92:214–217. doi: 10.1016/j.ijid.2020.01.050 32007643 PMC7110798

[17] Chan JFW, Yuan S, Kok KH, To KKW, Chu H, Yang J, et al. A familial cluster of pneumonia associated with the 2019 novel coronavirus indicating person-to-person transmission: a study of a family cluster. The Lancet. 2020;395(10223):514–523. doi: 10.1016/S0140-6736(20)30154-9 31986261 PMC7159286

[18] Phan LT, Nguyen TV, Luong QC, Nguyen TV, Nguyen HT, Le HQ, et al. Importation and human-to-human transmission of a novel coronavirus in Vietnam. New England Journal of Medicine. 2020;382(9):872–874. doi: 10.1056/NEJMc2001272 31991079 PMC7121428

[19] Kissler SM, Tedijanto C, Goldstein E, Grad YH, Lipsitch M. Projecting the transmission dynamics of SARS-CoV-2 through the postpandemic period. Science. 2020;368(6493):860–868. doi: 10.1126/science.abb5793 32291278 PMC7164482

[20] Wrapp D, Wang N, Corbett KS, Goldsmith JA, Hsieh CL, Abiona O, et al. Cryo-EM structure of the 2019-nCoV spike in the prefusion conformation. Science. 2020;367(6483):1260–1263. doi: 10.1126/science.abb2507 32075877 PMC7164637

[21] Huang Y, Yang C, Xu Xf, Xu W, Liu Sw. Structural and functional properties of SARS-CoV-2 spike protein: potential antivirus drug development for COVID-19. Acta Pharmacologica Sinica. 2020;41:1141–1149 doi: 10.1038/s41401-020-0485-4 32747721 PMC7396720

[22] Walls AC, Park YJ, Tortorici MA, Wall A, McGuire AT, Veesler D. Structure, function, and antigenicity of the SARS-CoV-2 spike glycoprotein. Cell. 2020;181(2):281–292 doi: 10.1016/j.cell.2020.02.058 32155444 PMC7102599

[23] Dai W, Zhang B, Jiang XM, Su H, Li J, Zhao Y, et al. Structure-based design of antiviral drug candidates targeting the SARS-CoV-2 main protease. Science. 2020;368(6497):1331–1335. doi: 10.1126/science.abb4489 32321856 PMC7179937

[24] Gordon DE, Hiatt J, Bouhaddou M, Rezelj VV, Ulferts S, Braberg H, et al. Comparative host-coronavirus protein interaction networks reveal pan-viral disease mechanisms. Science. 2020;370(6521):eabe9403 doi: 10.1126/science.abe9403 33060197 PMC7808408

[25] Li JY, Liao CH, Wang Q, Tan YJ, Luo R, Qiu Y, et al. The ORF6, ORF8 and nucleocapsid proteins of SARS-CoV-2 inhibit type I interferon signaling pathway. Virus research. 2020;286:198074. doi: 10.1016/j.virusres.2020.198074 32589897 PMC7309931

[26] Gordon DE, Jang GM, Bouhaddou M, Xu J, Obernier K, White KM, et al. A SARS-CoV-2 protein interaction map reveals targets for drug repurposing. Nature. 2020; 583:459–468 doi: 10.1038/s41586-020-2286-9 32353859 PMC7431030

[27] Chi X, Yan R, Zhang J, Zhang G, Zhang Y, Hao M, et al. A neutralizing human antibody binds to the N-terminal domain of the Spike protein of SARS-CoV-2. Science. 2020;369(6504):650–655. doi: 10.1126/science.abc6952 32571838 PMC7319273

[28] Letko M, Marzi A, Munster V. Functional assessment of cell entry and receptor usage for SARS-CoV-2 and other lineage B betacoronaviruses. Nature microbiology. 2020;5(4):562–569. doi: 10.1038/s41564-020-0688-y 32094589 PMC7095430

[29] Hoffmann M, Kleine-Weber H, Pöhlmann S. A multibasic cleavage site in the spike protein of SARS-CoV-2 is essential for infection of human lung cells. Molecular Cell. 2020;78(4):779–784.e5 doi: 10.1016/j.molcel.2020.04.022 32362314 PMC7194065

[30] Hyeonuk W, Sang-Jun P, Yeol KC, Taeyong P, Maham T, Yiwei C, et al. Developing a Fully-glycosylated Full-length SARS-CoV-2 Spike Protein Model in a Viral Membrane. The journal of physical chemistry B. 2020;124:7128–7137. doi: 10.1021/acs.jpcb.0c04553 32559081 PMC7341691

[31] Korber B, Fischer WM, Gnanakaran S, Yoon H, Theiler J, Abfalterer W, et al. Tracking changes in SARS-CoV-2 Spike: evidence that D614G increases infectivity of the COVID-19 virus. Cell. 2020;182(4):812–827. doi: 10.1016/j.cell.2020.06.043 32697968 PMC7332439

[32] Mahase E. Covid-19: What have we learnt about the new variant in the UK? BMJ. 2020;371. 33361120 10.1136/bmj.m4944

[33] Yurkovetskiy L, Wang X, Pascal KE, Tomkins-Tinch C, Nyalile TP, Wang Y, et al. Structural and functional analysis of the D614G SARS-CoV-2 spike protein variant. Cell. 2020;183(3):739–751.e8 doi: 10.1016/j.cell.2020.09.032 32991842 PMC7492024

[34] Aicardi F. Self-linking of spatial curves without inflections and its applications. Functional Analysis and Its Applications. 2000;34(2):79–85. doi: 10.1007/BF02482420

[35] Uribe-Vargas R. On singularities, “perestroikas” and differential geometry of space curves. Enseignement Mathematique. 2004;50(1/2):69–102.

[36] Spivak MD. A comprehensive introduction to differential geometry. vol. 5. Publish or perish; 1970.

[37] Hu S, Lundgren M, Niemi AJ. Discrete Frenet frame, inflection point solitons, and curve visualization with applications to folded proteins. Physical Review E. 2011;83(6):061908. doi: 10.1103/PhysRevE.83.061908 21797404

[38] Lundgren M, Niemi AJ, Sha F. Protein loops, solitons, and side-chain visualization with applications to the left-handed helix region. Physical Review E. 2012;85(6):061909. doi: 10.1103/PhysRevE.85.061909 23005129

[39] Lundgren M, Krokhotin A, Niemi AJ. Topology and structural self-organization in folded proteins. Physical Review E. 2013;88(4):042709. doi: 10.1103/PhysRevE.88.042709 24229215

[40] Hinsen K, Hu S, Kneller GR, Niemi AJ. A comparison of reduced coordinate sets for describing protein structure. The Journal of Chemical Physics. 2013;139(12):124115. doi: 10.1063/1.4821598 24089758

[41] Peng X, He J, Niemi AJ. Clustering and percolation in protein loop structures. BMC structural biology. 2015;15(1):22. doi: 10.1186/s12900-015-0049-x 26510704 PMC4625449

[42] Shapovalov MV, Dunbrack RL Jr. A smoothed backbone-dependent rotamer library for proteins derived from adaptive kernel density estimates and regressions. Structure. 2011;19(6):844–858. doi: 10.1016/j.str.2011.03.019 21645855 PMC3118414

[43] Liu J, Dai J, He J, Peng X, Niemi AJ. Can the geometry of all-atom protein trajectories be reconstructed from the knowledge of C *α* time evolution? A study of peptide plane O and side chain C *β* atoms. The Journal of chemical physics. 2019;150(22):225103. 31202245 10.1063/1.5082627

[44] Peng X, Chenani A, Hu S, Zhou Y, Niemi AJ. A three dimensional visualisation approach to protein heavy-atom structure reconstruction. BMC Structural Biology. 2014;27:14 doi: 10.1186/s12900-014-0027-8 25551190 PMC4302604

